# Primary glioblastoma of the cerebellum in a 19-year-old woman: a case report

**DOI:** 10.1186/1752-1947-6-329

**Published:** 2012-10-02

**Authors:** Moulay Rachid El El Maaqili, Adil Hossini, Nizar El Fatemi, Rachid Gana, Amar Saïdi, Mohammed Jiddane, Fouad Bellakhdar

**Affiliations:** 1 Department of Neurosurgery, Avicenne University Hospital, Lamfadel Cherkaoui Street, BP 6527, Rabat, Morocco; 2 Department of Anatomopathology, Avicenne University Hospital, Lamfadel Cherkaoui street, BP 6527, Rabat, Morocco; 3 Department of Neuroradiology, Avicenne University Hospital, Lamfadel Cherkaoui Street, BP 6527, Rabat, Morocco

**Keywords:** Glioblastoma, Posterior fossa, Magnetic resonance imaging, Surgery

## Abstract

**Introduction:**

Cerebellar glioblastoma is an uncommon adult lesion. The pathogeny and prognosis of cerebellar glioblastoma are still incompletely elucidated.

**Case presentation:**

We report the case of a 19-year-old Moroccan woman. A neurologic examination revealed the presence of cerebellar signs. A diagnosis of cerebellar glioblastoma is rarely made before surgery. An encephalic computer tomography scan and magnetic resonance imaging increased the accuracy of defining the nature of the lesion. Magnetic resonance imaging showed a heterogeneously enhancing mass in the vermis and the right cerebellar hemisphere. After surgery, glioblastoma was histologically confirmed. Post-operative radiotherapy followed. The outcome of our patient was favorable after one year of follow-up.

**Conclusions:**

We report an unusual case of cerebellar gliobastoma. Cerebellar glioblastoma multiforme should be considered in the differential diagnosis of a cerebellar mass lesion.

## Introduction

Glioblastoma multiforme (GBM) is the most common primary brain tumor in adults. It usually affects the cerebral hemispheres, and the peak age of onset is the sixth or seventh decade of life [1]. Cerebellar glioblastoma is a rare adult tumor. To the best of our knowledge, only a few cases have been reported [2,3]. The tumor represents 0% to 3.4% of primary GBMs [4,5]. In this case report, we present a case of primary cerebellar glioblastoma and discuss the physiopathology, clinical presentation, diagnosis, treatment, and general outcomes of this disease.

## Case presentation

**Figure 1:**
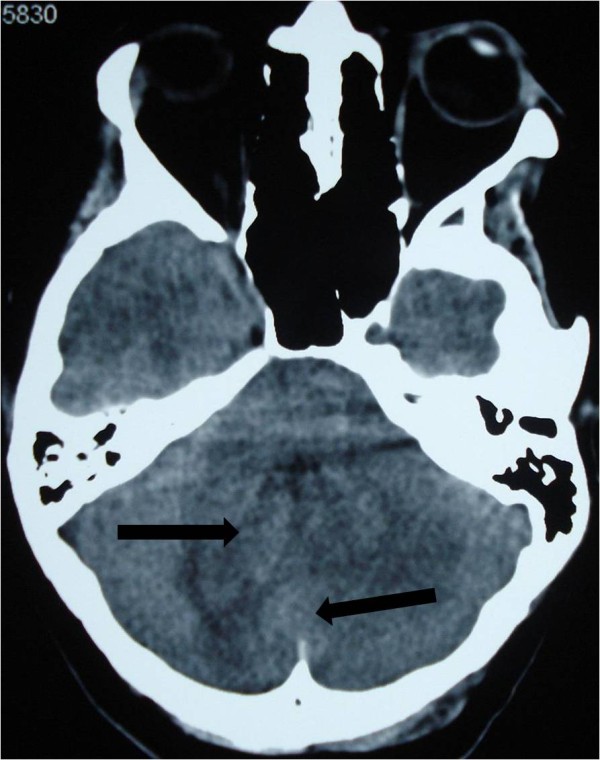
**Cerebral computed tomography scan of two lesions in the right cerebellar hemisphere and the vermis (arrow).** The lesions are spontaneously hypodense.

**Figure 2:**
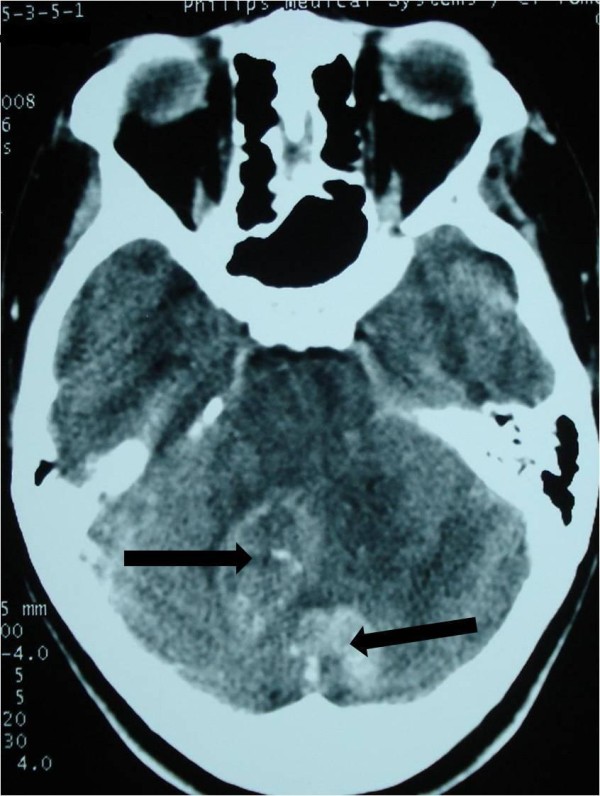
Vermis and right cerebellar lesions (arrow) with a peripheral heterogeneous enhancement after contrast injection and mass effect on the fourth ventricle.

**Figure 3:**
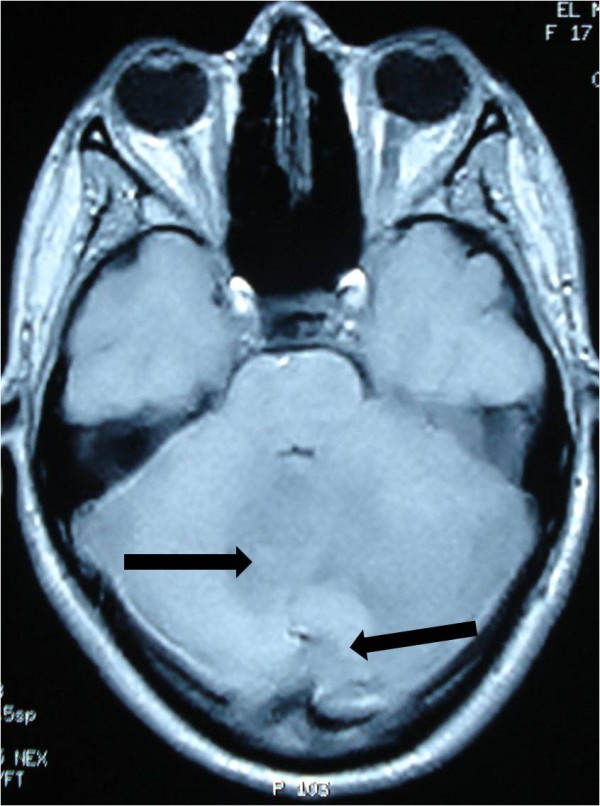
**Cranial magnetic resonance imaging of irregular contours of the mass lesions that are in contact with the fourth ventricle and the confluence of sinus (arrow).** The processes are heterogeneous on T1-weighted imaging.

**Figure 4:**
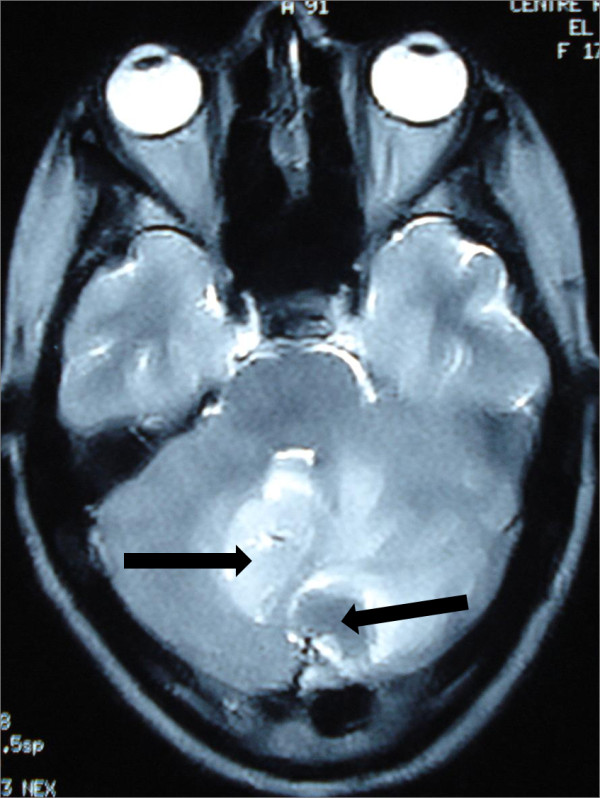
**The processes are heterogeneous on a T2-weighted image (arrow) with discrete surrounding edema.** The cerebellar lesion measures 3×4cm in size.

**Figure 5:**
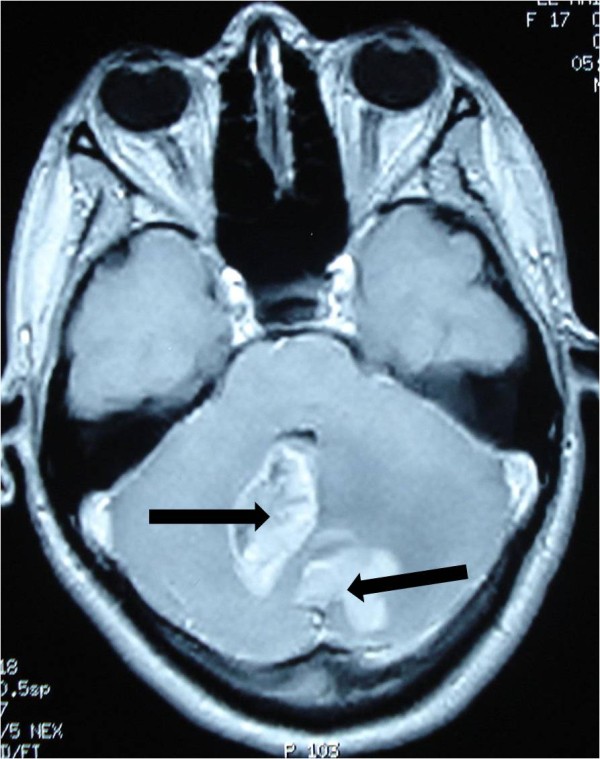
The lesions have a heterogeneous contrast ring enhancement with intratumoral necrotic central zones (arrow).

A 19-year-old Moroccan woman without any other medical history was admitted to our hospital with a two-month history of intracranial pressure with nausea, vomiting, and headache. A neurologic examination showed cerebellar signs, including cerebellar ataxia, dysmetria, and dysdiadochokinesia. The fundus oculi showed bilateral papillary edema. A cerebral computed tomography (CT) scan showed two lesions: one in the right cerebellar hemisphere and the other in the vermis. The lesions were spontaneously hypodense with a peripheral heterogeneous enhancement after contrast injection and mass effect on the fourth ventricle (Figures 1 and 2). Cranial magnetic resonance imaging (MRI) showed irregular contours of mass lesions. The processes were heterogeneous on T1- and T2-weighted images and this can be related to a subacute intratumoral hemorrhage with discrete surrounding edema. After gadolinium was administered, the lesion was 3×4cm in size and had heterogeneous ring enhancement and a central necrotic area (Figures 3, 4, and 5).

**Figure 6:**
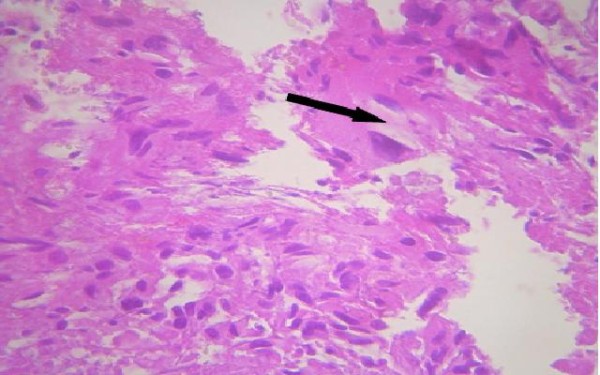
**Original coloration shows an intense cellular density.** The cells are characterized by a marked anisokaryosis. The nuclei are quite large. The cells have an abundant and eosinophilic cytoplasm (arrow). Numerous mitoses are observed. Stain: hematoxylin and eosin; magnification: ×400.

**Figure 7:**
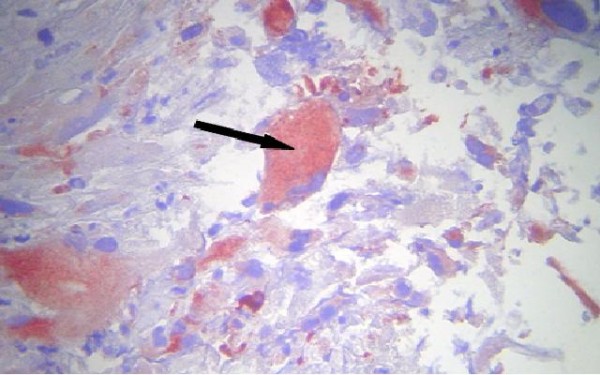
**Immunohistochemistry study by 3-amino-9-ethylcarbazole peroxidase method and antigenic restoration in warmth show a labeling of the cells with the anti-GFAP (anti-glial fibrillary acidic protein) antibodies.** The positivity of the GFAP confirmed the astrocytic nature of the lesion (arrow). An immunohistochemistry profile of glioblastoma is shown.

Our patient first had a ventriculoperitoneal shunt. On the second day, a suboccipital craniotomy was performed while our patient was in the prone position. After opening of the dura mater and corticectomy, the lesion was white, infiltrative, crumbly, and not very hemorrhagic, had no planes of cleavage, and had indistinct margins from adjacent normal white matter. A subtotal tumor resection was performed. A histopathological examination revealed a cellular tumor, which was consistent with glioblastoma (that met the World Health Organization criteria of grade 4 astrocytoma) and which was composed of elongated spindle-shaped cells with irregular, moderately pleomorphic and giant nuclei, and proliferative blood vessels and necrosis (hematoxylin and eosin stain (HES) ×100 and HES ×400). Immunohistochemistry was accomplished by means of the 3-amino-9-ethylcarbazole (AEC) peroxydase method and antigenic restoration, showing a marking of tumor cells with anti-GFAP (anti-glial fibrillary acidic protein) antibodies (Figures 6 and 7).

The post-operative course was uneventful, and the neurologic signs gradually improved. Our patient received post-operative radiotherapy to the posterior cranial fossa. There was no tumor recurrence after 12 months.

## Discussion

Glioblastoma, the most frequent tumor among all primary tumors of the central nervous system in adults, has a frequency of 50% [2,3,5]. Adult cerebellar glioblastoma is extremely rare, accounting for 0.24% to 3.8% of all intracranial glioblastomas [2-4,6,7]. From 1975 to 2011, 170 articles and abstracts about cerebellum glioblastoma were published, according to a search of the Medline database. The male-to-female ratio is 2:1 [8]. Cerebellar glioblastoma can be seen in all age groups. About 70% of lesions were seen in adults, and median age was 46.7 years; 30% were noted in children, and median age was 10.4 years [4,8]. As with our patient, localization is generally median or paramedian with a possible extension to the fourth ventricle [4]. The tumor is infiltrative and usually is localized in the deep white matter [6].

The pathogeny and prognosis of cerebellar glioblastomas are still incompletely elucidated because of their rarity [6]. Most authors have failed to fully explain the rarity of cerebellar GBM [1,4]. Based on the clinical and genetic data, there are two subsets of GBM [9]. Glioblastoma may develop *de novo* (primary type) or from previous low-grade astrocytomas (secondary type) [10]. Secondary glioblastomas develop more frequently in younger patients and often contain TP 53 mutation (65%), whereas primary glioblastomas affect mostly elderly patients and generally are characterized by the loss of heterozygosity 10 q (LOH 10q) (70%), epidermal growth factor receptor amplification (36%), and TP 53 mutation at a frequency of lower than 30% [10]. Radiotherapy was also incriminating for the possibility of anaplasic progression of mature cerebellar astrocytic cells [6,11]. Maat-Schieman *et al*. [11] reported a case of vermis cerebellar astrocytoma that developed after radiotherapy for craniopharyngioma. Other authors reported the development of glioblastoma after radiotherapy for pilocytic astrocytoma, medulloblastoma, and other posterior fossa tumors [12]. Our patient had no history of radiotherapy.

Symptomatology of cerebellar glioblastoma, like that of any other cerebellar tumor, generally includes headache, gait disturbance, vertigo, nausea, vomiting, and ataxia (as in our case). Indeed, dizziness, neck pain, and mental confusion can be present [2,4,7]. These symptoms are nonspecific and can occur in many diseases.

The diagnosis of cerebellar glioblastoma is rarely made before surgery, even though CT scan and especially MRI have increased the accuracy of defining the nature of the lesion [2,3,6]. MRI shows decreased T1- and increased T2-weighted signal intensities compared with white matter. In the majority of cases, a peripheral heterogeneous enhancement after contrast injection, irregular contours, and a central necrosis similar to that of supratentorial glioblastoma are noted. Intratumoral hemorrhage is suggested by mixed signals on T1- and T2-weighted signals [2,3,6]. MRI diffusion/perfusion and MRI spectroscopy examinations can also facilitate the characterization of the lesions and the differential diagnosis [2].

Proton spectroscopy of high-grade gliomas shows a significant reduction in NAA (N-acetyl aspartate), owing to the loss in neuronal elements, and a choline peak elevation that may reflect increased membrane synthesis. The presence of a lactate peak correlates with tumor hypoxia [2,3,6,13]. Hydrocephalus was reported in our patient and also in other cases. It was found in four out of nine patients of Kuroiwa *et al*. [4].

Cerebellar metastases are the most frequent posterior cerebral fossa tumors for adults and represent the most common differential diagnosis, particularly if the lesion is solitary and there is no history of primary lesion [2,4,6]. However, the margins of metastases are distinct with a most important surrounding edema. Metastasis may demonstrate cystic components or show hypointensity on T2-weighted images, suggesting intratumoral hemorrhage, calcification, or mucinous components, while GBM grows infiltratively with peripheral heterogeneous enhancement and poorly defined margins [4]. Peritumoral edema is rather mild compared with that of metastatic tumors [1,7]. Intratumoral hemorrhage is common, but calcification or cystic components are rarely seen.

Diagnostic difficulty also arises when there are several lesions, as with our patient. Other causes like abscesses, granulomas, or metastases should be considered, also primitive neuroectodermal tumor of the posterior fossa, medulloblastoma, ependymoma, pilocytic astrocytoma, or hemangioblastoma [1,2,4]. As the posterior fossa is a small compartment, it is very difficult to be absolutely certain that these two lesions were completely distinct. They were probably two focal areas of tumor connected with each other. As it was not possible to resect both lesions, there was no evidence to prove the true multifocal nature of this tumor.

The treatment of cerebellar glioblastoma is usually palliative and encompasses surgery, radiotherapy, and chemotherapy [1]. The decision regarding surgery for glioblastoma depends on the patient’s age, performance status, proximity to “eloquent” areas of brain, feasibility of decreasing the mass effect, resectability of the tumor, and, in a patient with recurrent disease, the time since previous surgery. Surgical treatment includes stereotactic or open biopsy and debulking with subtotal or gross resection. Surgery will achieve a diagnosis, decrease intracranial pressure, increase survival, and reduce the need for corticosteroid therapy [1,3,4].

After surgery, the patient can be treated with fractionated external beam radiotherapy [3,7]. Total doses of 54 to 60Gy in 1.8 to 2.0Gy fractions are administered to the gross tumor volume [1]. Craniospinal irradiation can be recommended for children because of the high incidence of cerebrospinal fluid dissemination of cerebellar GBM [7]. The chemotherapy of choice is temozolomide, although bis-chloronitrosourea, lomustine, or PCV (procarbazine, lomustine, vincristine) can also be used [14]. Patients with a good performance score should be treated with daily temozolomide (75mg/m^2^) administered with post-operative radiation therapy followed by one year of a dose of 150 to 200mg/m^2^ per day for five days each month [15].

The biological behaviors of cerebellar and supratentorial GBM are similar. On the other hand, the prognosis of cerebellar glioblastoma for younger patients is similar to that of anaplasic astrocytoma and probably has a better outcome than cerebral glioblastoma [3]. The mean survival of cerebellar glioblastoma after the beginning of the symptomatology has been reported to be approximately 12 to 19 months [3,7,8].

## Conclusions

Cerebellar GBM is rare and should be considered in the differential diagnosis of a cerebellar mass lesion. MRI can facilitate the characterization of the lesions and the differential diagnosis, particularly from metastases. Aggressive surgical treatment along with radiation therapy remains the established management strategy.

## Consent

Written informed consent was obtained from the patient for publication of this case report and any accompanying images. A copy of the written consent is available for review by the Editor-in-Chief of this journal.

## Abbreviations

CT: Computed tomography; GBM: Glioblastoma multiforme; HES: Hematoxylin and eosin stain; MRI: Magnetic resonance imaging.

## Competing interests

The authors declare that they have no competing interests.

## Authors’ contributions

AH wrote and revised the manuscript and provided comments. MJ prepared the figures and provided comments. AS prepared the biopsy and provided comments. My R El M revised the manuscript and provided comments. NF revised the manuscript and provided comments. RG revised the manuscript and provided comments. FB revised the manuscript and provided comments. All authors read and approved the final manuscript
